# Modified Farmer's Flap for Cosmetic Correction of Post-burn Small Finger Deformity: A Case Report

**DOI:** 10.7759/cureus.106550

**Published:** 2026-04-06

**Authors:** Murali K Manikkavelu, Sara Almaradweh, Menar Wahood

**Affiliations:** 1 General Surgery, American University of Antigua, St. John's, ATG; 2 Medicine, Avalon University School of Medicine, Willemstad, CUW; 3 Surgery, Northwestern Medicine Regional Medical Group, Orland Park, USA

**Keywords:** burn contracture, contracture release, cosmetic reconstruction, farmer’s flap, hand surgery, local rotational flap, pediatric burn, reconstructive surgery, small finger deformity, soft tissue reconstruction

## Abstract

Childhood burn injuries can lead to persistent digital contractures and contour deformities that affect both function and psychosocial well-being. We report the case of a 17-year-old female patient with a longstanding post-burn contracture and cosmetic deformity of the small finger after a burn sustained at age two, initially treated conservatively with compression dressings. Years later, she presented with residual soft tissue atrophy and tethering with clinically apparent foreshortening and expressed concern primarily about the cosmetic appearance. A modified Farmer’s flap, traditionally described for hallux varus correction of the great toe, was adapted as a local rotational flap to address the soft tissue deficiency and restore contour after scar release. The procedure improved the overall appearance and contour of the small finger with a satisfactory cosmetic match using local tissue. At postoperative follow-up, the patient reported satisfaction with the aesthetic outcome; a decrease in small-finger range of motion was anticipated and accepted as a trade-off, and no complications were observed. This case demonstrates that Farmer’s flap principles may be adapted for selected post-burn hand deformities when cosmetic restoration is a primary goal and local tissue rearrangement is appropriate.

## Introduction

Burn injuries in pediatric patients can have lasting physical and psychological effects, particularly when scarring results in visible disfigurement. Beyond the functional limitations caused by scarring and contracture, pediatric burn survivors may experience persistent psychosocial stressors, including anxiety and traumatic stress symptoms, altered self-esteem, and social distress, all of which can negatively affect quality of life [1]. Because of these factors, the perceived success of reconstruction is often shaped not only by functional recovery but also by appearance-related concerns and social confidence. In addition, burn injury can affect the family unit; parental stress and post-traumatic stress symptoms may interact with a child’s distress and contribute to overall well-being and recovery, further influencing expectations and satisfaction with long-term outcomes [2].

The Farmer’s flap is a local rotational flap originally described for correction of congenital hallux varus, a medial deviation deformity of the great toe [3]. In the foot surgery literature, Farmer’s technique is discussed as a local soft-tissue rearrangement method in which tissue from the first web space is mobilized and rotated to address medial soft-tissue deficiency after correction, helping restore contour and soft-tissue balance while providing well-vascularized coverage. Subsequent descriptions and modifications emphasize using adjacent supple tissue to cover the medial defect while protecting local neurovascular structures [4]. Although this method is well established in foot reconstruction, its principles have been primarily described in the context of hallux varus and are less commonly discussed in hand reconstruction, despite similar needs for reliable local coverage and contour restoration.

Post-burn hand deformities commonly involve contracture, tethering, and contour loss that affect both appearance and function; recently published reviews report flaps as the most commonly used reconstructive option after release [5]. In the hand, even subtle contour changes can be highly noticeable, and rearranging adjacent local tissue to restore contour after release can meaningfully improve overall appearance in addition to function. We present a case in which a modified Farmer’s flap was adapted to address a post-burn cosmetic deformity of the small finger, illustrating how the principles of local tissue rearrangement and soft tissue balancing may be applied beyond the foot.

## Case presentation

A 17-year-old right-handed girl presented to the orthopedic hand surgery clinic for evaluation of a longstanding deformity of the right small finger. The deformity dated back to a burn injury sustained at two years of age, which was managed nonoperatively with compression dressings at the time, without surgical intervention, as reported by the patient. Over the ensuing years, she developed a persistent scar-related deformity of the small finger with a visible contour abnormality, prompting evaluation as an adolescent for possible correction.

At presentation, the patient identified cosmetic appearance as her primary concern. She was bothered by the visible deformity of the right small finger and sought improvement in contour and overall appearance. She did not report specific functional complaints at the time of presentation; her primary concern was cosmetic appearance.

**Figure 1:**
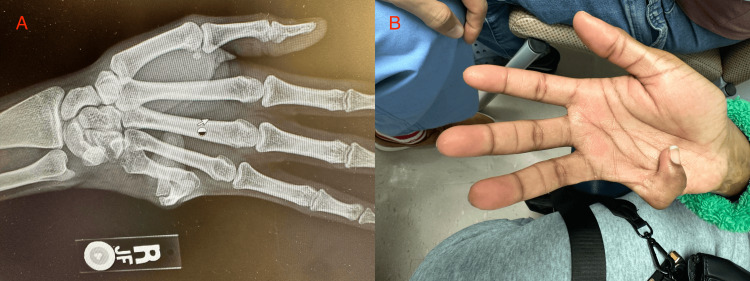
Preoperative evaluation of the right small finger. (A) Preoperative radiograph demonstrating no osseous abnormality. (B) Preoperative clinical photograph (palmar view) demonstrating flexion contracture and visible deformity of the small finger.

The patient was clinically stable with vital signs within normal limits, and the remainder of the physical examination was unremarkable. Assessment of the right hand demonstrated a scar-related deformity of the small finger with flexion contracture and tethered soft tissue, producing a visible cosmetic abnormality. The digit appeared clinically foreshortened, consistent with burn-related soft tissue deficiency and contracture. Radiographs demonstrated no osseous abnormality, suggesting that the deformity was related primarily to soft tissue contracture rather than bony pathology (Figures 1A, 1B).

Operative and nonoperative options were discussed with the patient, with counseling focused on her primary goal of cosmetic improvement and the potential trade-off between aesthetic restoration and postoperative small-finger range of motion. After shared decision-making, she elected to proceed with operative management. Given the presence of scar-related tethering and contour deficiency, a local tissue rearrangement approach was selected to improve contour using adjacent, well-perfused tissue. Flap-based reconstruction is also the most commonly reported reconstructive option following contracture release in recently published reviews [5]. A modified Farmer’s flap, classically described for hallux varus correction, was chosen as a local rotational flap concept to achieve contour restoration and coverage with an expected favorable cosmetic match [3,4].

Surgical technique

**Figure 2:**
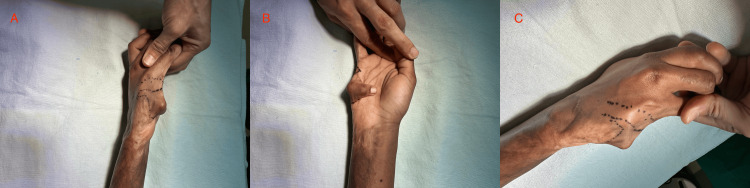
Preoperative flap design and skin markings. (A) Lateral (ulnar) view demonstrating the outlined triangular local flap along the dorsoulnar aspect of the right small finger with a broad proximal base. (B) Palmar view demonstrating the planned markings along the ulnar border of the hand at the base of the small finger. (C) Dorsal-oblique view demonstrating the overall configuration of the planned flap and incision markings over the dorsoulnar small-finger/ulnar-hand region.

In the operating room, the scar-related deformity and areas of tethering were assessed. A triangular local flap was planned along the dorsoulnar aspect of the small finger with extension onto the adjacent ulnar hand. The flap was designed with a broad proximal base to support reliable perfusion and allow rotation toward the area of greatest contour deficiency, as shown by the intraoperative markings (Figures 2A-2C).

The flap was elevated and mobilized with careful soft-tissue handling. Dissection and flap mobilization were performed with attention to protecting the digital neurovascular bundles and maintaining flap perfusion throughout. After release of the scar tethering, the flap was rotated from the dorsoulnar surface and advanced toward the volar aspect to redistribute local tissue and improve contour over the region of greatest deficiency.

**Figure 3:**
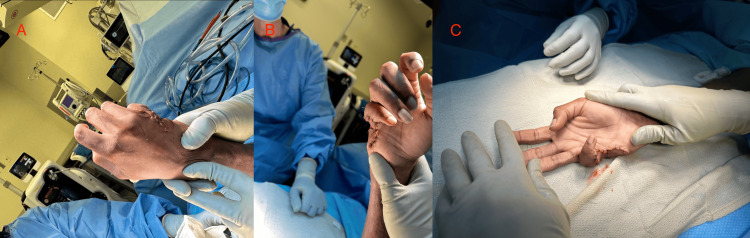
Immediate postoperative appearance following flap inset and closure. (A) Dorsal view at the conclusion of the procedure demonstrating the incision closure and flap inset along the ulnar aspect of the hand. (B) Palmar view at the conclusion of the procedure demonstrating closure and improved contour of the small finger/ulnar border. (C) Oblique close-up view demonstrating the immediate postoperative appearance of the flap inset and closure along the ulnar aspect of the small finger.

The flap was inset without excessive tension, and the donor site was closed primarily. Immediate postoperative assessment demonstrated improved contour and a more favorable cosmetic appearance of the small finger at the conclusion of the procedure (Figures 3A-3C).

**Figure 4:**
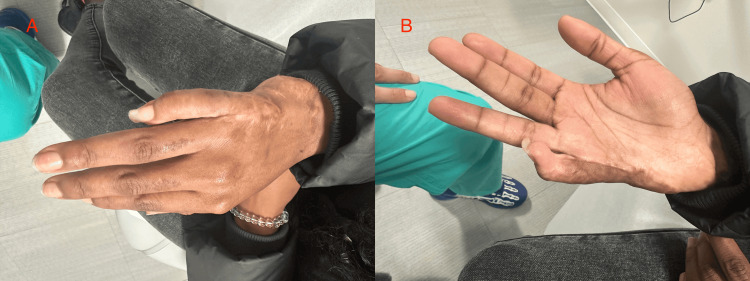
Postoperative appearance at follow-up. (A) Dorsal view demonstrating improved contour of the right small finger and ulnar hand following flap reconstruction. (B) Palmar view demonstrating improved soft-tissue contour along the ulnar aspect of the right small finger at follow-up.

At follow-up, the patient demonstrated improved contour and overall cosmetic appearance of the right small finger. She reported satisfaction with the aesthetic result. Motion remained limited, consistent with the pre-existing contracture and the expected trade-off of the reconstruction. No postoperative complications were observed, and the contour improvement was maintained (Figures 4A, 4B).

## Discussion

The Farmer flap was first described by A.W. Farmer in 1958 for surgical correction of congenital hallux varus, a condition in which medial deviation and soft tissue deficiency can make alignment and coverage difficult [6]. In the classic toe literature, the Farmer procedure is described as a rotational skin and subcutaneous flap used to redistribute tension, improve medial soft tissue coverage, and assist with restoring alignment, often combined with other corrective steps depending on the deformity [3,4,6]. Variations of the Farmer procedure and related modifications have continued to appear in the foot literature, including reports describing its use for coverage in hallux varus reconstruction [3,4]. Despite this established role in the foot, we did not identify published reports describing the use of a Farmer-type flap in the hand, making this case a novel adaptation of a long-standing reconstructive concept.

Post-burn contractures of the hand are challenging because treatment must address both scar release and durable resurfacing while preserving as much function and contour as possible [7-9]. Surgical options exist along a spectrum, ranging from scar excision with skin grafting to local tissue rearrangement such as Z-plasties and local flaps when adequate surrounding tissue is available [5,7,10]. In situations where local options are insufficient, reconstruction may require more extensive tissue transfer, including distant pedicled flaps or free flaps, particularly when deeper structures are at risk or when coverage demands exceed what local rearrangement can provide [7,9]. Glove-like abdominal flaps have been reported as one method to address contractures involving the dorsal hand and fingers, illustrating how distant tissue can be used when local solutions are limited [8]. Although free flaps are versatile and can achieve reliable coverage, they may involve greater operative complexity, postoperative monitoring, and a higher burden of potential complications compared with simpler local procedures in selected cases [9]. In our patient, adapting the Farmer design provided a technically straightforward local flap option that was capable of improving contour in a focused deformity while avoiding the complexity of microsurgical reconstruction [7,10].

Several practical advantages were observed in this case. Because the reconstruction relied on adjacent local tissue, the flap provided a better match in skin quality and durability than grafted skin, which can be prone to contour irregularity and may be less robust in areas exposed to daily mechanical stress [5,7,10]. The donor area was closed primarily, minimizing additional morbidity, and the reconstruction avoided the time, monitoring requirements, and complication profile typically associated with microsurgical free tissue transfer [9]. Overall, the flap provided a single-stage solution that aligned with the patient’s primary goal of cosmetic contour improvement.

The limitations in this case included a reduced range of motion of the small finger, attributable to the bulk of the reconstruction and rearrangement of local soft tissue. Given the pre-existing contracture and the patient’s priority of cosmetic contour, this tradeoff was anticipated, discussed preoperatively, and considered acceptable. Additional limitations include restricted applicability when surrounding skin is extensively scarred, the possibility of residual bulk or contour irregularity, and the potential for recurrent contracture over time, all of which reflect broader principles in contracture reconstruction where technique selection must be individualized to scar pattern, local tissue quality, and patient priorities [7,10]. These considerations underscore that this approach should be viewed as a selective option for localized deformities rather than a universal solution for extensive contractures [10].

Finally, this case adds to the range of reconstructive approaches available for burn-related hand deformities. In younger patients, appearance can carry meaningful psychosocial impact, and reconstructive choices that improve contour and visible deformity may have value beyond purely functional outcomes [1,2]. While this report describes a single patient, the favorable cosmetic result supports further exploration of this technique in carefully selected cases, with longer follow-up and broader evaluation of patient-reported outcomes to better define durability and appropriate indications [10].

## Conclusions

This case demonstrates that a modified Farmer flap concept, traditionally described for hallux varus correction in the great toe, can be adapted for local tissue transfer in the hand to improve contour in a selected postburn small-finger deformity. In this case, the approach provided a favorable cosmetic match using adjacent tissue, with limited motion accepted as a trade-off based on the patient’s goals. Further experience and longer follow-up are needed to better define durability, functional outcomes, and appropriate indications.
